# Seagrass contribution to blue carbon in a shallow karstic coastal area of the Gulf of Mexico

**DOI:** 10.7717/peerj.12109

**Published:** 2021-09-10

**Authors:** Tania C. Cota Lucero, Jorge A. Herrera-Silveira

**Affiliations:** Departamento Recursos del Mar, Centro de Investigación y de Estudios Avanzados (CINVESTAV) del Instituto Politécnico Nacional Unidad Mérida, Mérida, Yucatán, México; Laboratorio Nacional de Resiliencia Costera (LANRESC), Sisal, Yucatán, México

**Keywords:** Autochthonous, Blue carbon, Mangroves, Seagrasses

## Abstract

Seagrass meadows provide multiple ecosystem services, including carbon sequestration. However, seagrass meadows are among the most threatened ecosystems worldwide. Determining the magnitude of the carbon stocks in seagrass meadows at the regional scale allows for the estimation of their global magnitude and identification of their importance in regional environmental mitigation strategies. The objective of the present study was to determine the structure of seagrass meadows in the Los Petenes Biosfera Reserve (LPBR) and evaluate their contributions to sinks of carbon in this system, located in Yucatan, which is considered the region with the largest seagrass extension in Mexico. Analyses of the seagrass meadows were executed following standardized protocols (spectral analysis, and isotope and carbon stock analyses). The LPBR stores an average of 2.2 ± 1.7 Mg C ha^−1^ in living biomass and 318 ± 215 Mg C ha^−1^ in sediment (top 1 m), and this carbon stock decreases with water depth. The seagrass community extends 149,613 ha, which represents the largest organic carbon stock (47 Tg C) documented in seagrass meadows in Mexico. Macroalgae and seagrass represent 76% of the organic carbon stored in sediment. If LPBR seagrass meadows are lost due to natural or anthropogenic impacts, 173 Tg CO_2eq_emissions could be released, which corresponds to the emissions generated by fossil fuel combustion of 27% of the current Mexican population. This information emphasizes the importance of seagrass meadows as a carbon sink in the region and their contribution to climate change mitigation, thus allowing for the implementation of necessary conservation strategies.

## Introduction

Seagrasses provide numerous ecosystem services, such as serving as a habitat and refuge for a high diversity of species, retaining sediment and protecting the coastal zone, healthy seagrass meadows improve water quality, and regulate the impacts of greenhouse gases, particularly CO_2_ (Hemminga & Nieuwenhuize, 1990; Costanza et al., 2014). Indeed, seagrasses are more efficient than tropical forest in carbon sequestration (McLeod et al., 2011).

Half of the carbon buried in marine sediments is found in coastal blue carbon habitats combined (*i.e.,* mangrove, salt marshes and seagrass) (Duarte, Sintes & Marbà, 2013). Seagrass have a larger distribution (0.33 × 10^6^ km^2^) than salt marshes and mangroves, whose habitats are restricted to intertidal areas along the coast (Charpy-Roubaud & Sournia, 1990; Duarte & Cebrián, 1996; Short et al., 2007). Because of their wide distribution and high productivity, it is important to quantify the contribution of seagrass meadows to continent-scale carbon budgets (Duarte & Chiscano, 1999; Laffoley & Grimsditch, 2009; Nellemann et al., 2009).

Shoot density, leaf area, and the specific characteristics of seagrass species regulate carbon storage (Mazarrasa et al., 2018). The structural complexity of seagrasses is related to both the above- and belowground biomass and contributes to sediment retention processes by trapping particles accumulating in sediments for millennia in the form of organic carbon (Hendriks et al., 2008; McLeod et al., 2011; Duarte & Krause-Jensen, 2017). Carbon stored in seagrass sediments comes from both the detritus produced by the degradation of its biomass (leaves, rhizomes, and roots), considered autochthonous carbon (Agawin & Duarte, 2002; Hendriks et al., 2008; Tanaya et al., 2018), and from allochthonous sources such as the contributions of river sediments (Signa et al., 2017; Macklin et al., 2019). The origin of the carbon in seagrass ecosystems differs at the regional level. In some areas the proportion of allochthonous carbon in seagrass meadows exceeds autochthonous carbon (Gacia, Duarte & Middelburg, 2002; Bouillon et al., 2008); these differences indicate the degree of connectivity between seagrass meadows and other ecosystems.

Anthropogenic impacts threaten seagrasses worldwide and have caused the disappearance of these habitats (Orth et al., 2006; Waycott et al., 2009). These impacts must be monitored at the regional scale (Carmen et al., 2019), to improve: (1) estimates of carbon stocks and the contribution of seagrass to global carbon stocks (Serrano et al., 2014); (2) relate carbon sources and their ability to store organic carbon with the influence of groundwater; and (3) provide data on the contribution of autochthonous *vs* allochthonous materials in carbon stocks.

Few studies have quantified organic carbon in seagrass ecosystems in Mexico, or related habitat health and the structure of these meadows (Samper-Villarreal et al., 2016; Herrera-Silveira et al., 2020). In this context, it is important to determine the carbon stocks both in seagrass biomass and sediments associated with these habitats, as well as their sources, the structural complexity of the seagrass, and the water quality. Here, we hypothesized that the seagrass carbon stocks and their contribution to sediments differ with water depth and that habitat-forming seagrass is the main source of the accumulated organic matter in associated sediments.

This may help improve carbon estimations on a global scale and the variables associated with those stocks. The study area corresponds to the marine protected area of Los Petenes Biosphere Reserve (LPBR), in the Gulf of Mexico. This region, of 151,200 ha (Pérez-Espinosa et al., 2019), is one of the largest continuous extension of seagrass on the Mexican coast. Therefore, the primary objective of the present study was to characterize the structural complexity of seagrass meadows and determine their relationship with the spatial distribution of organic carbon stocks along to environmental gradients related to water depth. A secondary objective was to identify the main sources of this carbon and the influences of environmental variables.

## Materials & Methods

### Study area

**Figure 1:**
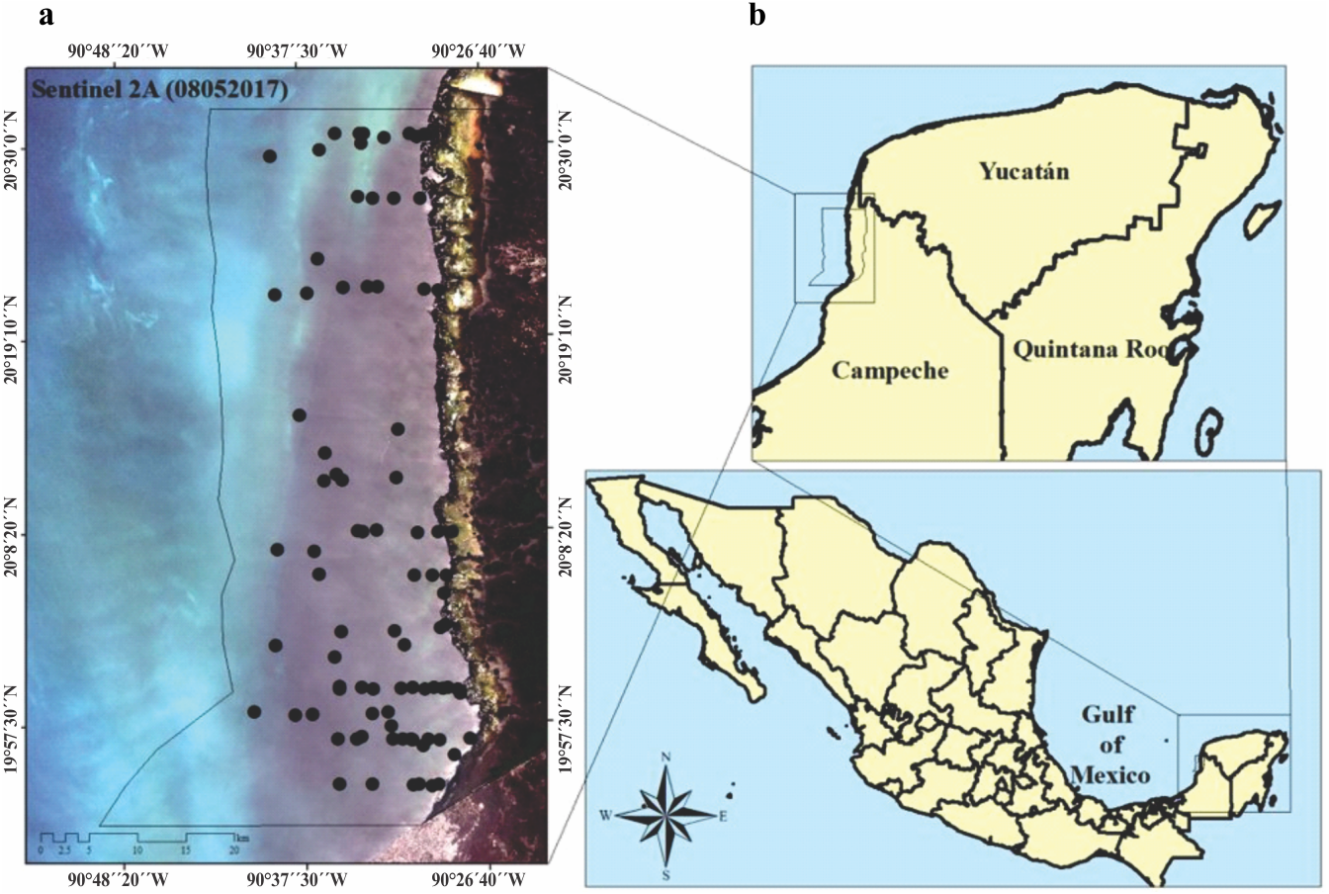
(A) Study area, imagen showing the location of seagrass (black circles), preprosed Sentinel 2A image. Image was acquired on May 8, 2017 (Path/Row: 21/46) pixel size 10 m. Black line (LPBR limits). (B) Los Petenes Biosphere Reserve (LPBR), in the Gulf of Mexico in Campeche, México.

The LPBR is one of the largest marine reserves (282,857 ha) in the southeast region of the Gulf of Mexico (Fig. 1). Mangroves and petenes (vegetation islands similar to hammock ecosystems in Florida, USA) cover 23% of the terrestrial surface. The coastal zone is estuarine (181,991 ha), showing a substrate enriched in carbonates due to the karst characteristics of the continental shelf in this region. The largest extension of seagrasses in Mexico is distributed in this protected area, and the community is composed of three species (*Thalassia testudinum*, *Syringodium filiforme* and *Halodule wrightii*) that have been recorded at depths of up to 5 m. This community shares substrates with a large diversity of macroalgae (Mateo-Cid et al., 2013). This region maintains strong coastal hydrological connectivity since its karst nature favors groundwater discharges to the coast, thus contributing to the supply of nutrients there (Grivel-Piña, 1992; CONAGUA, 2006). Along its 100 km coastline, there are only two communities of less than 1000 inhabitants each, so it is an area of low anthropic environmental impact. This region is an area of biological, ecological, and scientific importance as a result of its conservation and diversity (Corbalá, Río & García, 2007).

### Water quality

We visited the LPBR in May, 2017 and 2018 under CONANP permission (F.OO.9. DRPYyCM/060/2021). Transects were established perpendicular to the coast traveling ∼25 km considering a depth gradient of 1 to 5 m (Fig. 1). At sampling stations, physicochemical water quality data and seagrass samples were collected. Water depth was measured using a portable depth gauge (Hondex Ps-7); temperature, salinity, and dissolved oxygen (OD) were measured *in situ* using a YSI-2030 multiparameter probe at 50 cm depth intervals from the surface to the bottom. Incident light data (%) were obtained using a LI-250A spherical sensor (LICOR) that collected measurements every 50 cm deep across the entire water column. Water samples were collected in the immediate vicinity of the seagrass meadows for dissolved inorganic nutrient analysis (*n* = 79). The nutrients analyzed were nitrates + nitrites (NO_3_^−^ + NO_2_^−^), soluble reactive phosphorus (SRP), ammonium (NH_4_^+^), and soluble reactive silica (SRSi). These analyses were performed according to the standard methods described in Strickland & Parsons (1972). The concentration of chlorophyll-a (*Chl-a*) was also determined using the method of Parsons, Maita & Lalli (1984). From these environmental variables, the trophic index “TRIX” was calculated (Vollenweider et al., 1998; Melaku, Solidoro & Umgiesser, 2003; Jorgensen et al., 2005).

### Seagrass community characterization

The extension of the seagrass meadows in this protected marine area was determined using a Sentinel 2A multispectral image, which has a pixel size of 10 m and a radiometric resolution of 12 bits. This image corresponds to May 8, 2017 (Path/Row: 21/46) (European Space Agency, 2018), in which the image composition was generated by means of the blue (490 nm), green (560 nm), and red (665 nm) bands. Once this image composition was obtained, ground masking and radiometric correction (DOS-Dark Object Subtraction) were performed (Chavez Jr, 1988). For the classification of the image, four classes were considered based on an analysis of conglomerates with 80% similarity. From these classes and field verifications of 117 sites, spectral signatures of four types of seagrass beds were generated (coverage%, Table 1, Supporting Information): monospecific meadows of *Thalassia testudinum* (TtMa), mixing seagrass meadows (*T. testudinum, S. filiforme, H. wrightii*) with macroalgae (MxMa), mixing meadows dominated by *Syringodium fliforme* (MxSf), and *Syringodium filiforme* and *Thalassia testudinum* beds (SfTt). In the latter class, just one sample with *H. wrigthii* was found (Supplementary material, Table 1). The supervised classification was carried out by means of the maximum likelihood estimation. To determine the accuracy of the classification, we determined the Kappa statistic, which measures the level of agreement between the classes (Song et al., 2001). Sampling campaigns were designed to collect data and samples at several stations according to the area of each type of seagrass meadow. The characterization of the seagrass beds consisted of determining coverage (%) and density, following the Seagrass-Watch percent cover standard (McKenzie, 2003), and they were established at each station using a random scheme. Duplicate samples of seagrass biomass (leaves, rhizomes, and roots) were collected at each station using the standing crop method with a 15 cm diameter core (CARICOMP, 2001). In the laboratory, biomass samples were cleaned and epiphytes were removed. Once cleaned, samples were subdivided into two components (aboveground and belowground) and dried at 70 °C until a constant weight was obtained. Specific morphometric shoot variables evaluated were the maximum length of the leaves of each species, the width of the leaf and number of leaves per shoot (only for *T. testudinum*), and the leaf area index (LAI), which was calculated for *T. testudinum* (Bulthuis, 1990). To estimate the average carbon content in the biomass (C_bio_) of above- and belowground seagrass components, the values of the dry weights (g Dw m^−2^) were converted to carbon equivalents using conversion factors for each species: *Thallasia testudinum* (0.36), *Syringodium filiforme* (0.32), and *Hallodule wrightii* (0.34) (Short et al., 1985; Fourqurean & Zieman, 2002), Papiol et al., *unpublished data*). Afterwards, these values were finally extrapolated to Mg C ha^−1^.

**Table 1:** Summary of the distribution of the water quality variables. Summary of water quality parameter measured in LPBR at different water depths (1–5 m depth), showing mean ± SD, median, minimum and maximum values. Statistical data test (ANDEVA and Kruskall-wallis test). Statistical differences are indicated in bold.

Depth	1 m	2 m	3 m	4 m	5 m	gl	Critical value	*P*
**Temperature(°C)**						84	*F* = 0.85	0.497
Mean ± SD	29.11 ± 0.51	28.60 ± 0.31	29.23 ± 0.24	28.44 ± 0.20	29.06 ± 0.68			
Median	28	28	29	28	28			
Min–Max	28–32	27–31	27–34	27–30	27–39			
**Sal (ups)**								
Mean ± SD	36 ± 4.1	37 ± 2	36 ± 2.4	35 ± 1.7	34 ± 1.3	**4**	**H=16.19**	**0.003**
Median	37	38	36	35	35			
Min–Max	28–41	34–40	31–41	31–37	31–35			
**OD (mg l** ^−1^ **)**						**4**	**H=17**	**0.002**
Mean ± SD	8.67 ± 0.73	6.73 ± 0.47	7.03 ± 0.15	6.31 ± 0.30	6.31 ± 0.18			
Median	9	7	7	6	6			
Min–Max	5–12	3–9	5–9	3–8	5–8			
**NO**_2−_ **+ NO**_3−_ **(µmol l** ^−1^ **)**						76	*F* = 2.04	0.098
Mean ± SD	2.37 ± 0.73	6.13 ± 2.03	7.53 ± 1.56	4.53 ± 1.79	1.73 ± 0.20			
Median	2.19	2.08	2.91	1.77	1.67			
Min–Max	0.66–7.23	0.37–19.23	0.05–26.14	0.75–25.74	0.76–3.30			
**SRP (µmol l** ^−1^ **)**						78	*F* = 0.19	0.943
Mean ± SD	0.35 ± 0.08	0.32 ± 0.04	0.36 ± 0.04	0.38 ± 0.08	0.38 ± 0.08			
Median	0.28	0.3	0.3	0.3	0.22			
Min–Max	0.15–0.70	0.10–0.60	0.11–0.80	0.11–1.16	0.10–0.81			
**NH**_4+_ **(µmol l** ^−1^ **)**						**78**	**F=3.7**	**0.008**
Mean ± SD	1.38 ± 0.27	1.24 ± 0.23	1.51 ± 0.13	1 ± 0.20	0.64 ± 0.09			
Median	1.47	1.14	1.44	0.71	0.58			
Min–Max	0.24–2.66	0.18–3.48	0.27–3.14	0.28–3.38	0.14–1.21			
**SRSi (µmol l** ^−1^ **)**						80	*F* = 1.75	0.146
Mean ± SD	38.94 ± 8.58	15.95 ± 3.18	25.19 ± 4.31	17.94 ± 6.85	14.09 ± 9.07			
Median	31.69	12.54	19.73	7.97	4.16			
Min–Max	8.10–75.13	3.88–48.22	2.90–82.94	0.42–112	0.33–121.7			
**Cl** ***a*** **(µg l** ^−1^ **)**						33	*F* = 0.59	0.667
Mean ± SD	3.41 ± 0.59	2.85 ± 1.47	2.88 ± 1.19	3 ± 1.12	1.59 ± 0.32			
Median	3.22	1.36	1.82	1.33	1.49			
Min–Max	1.91–5.15	1–11.65	0.5–8.23	0.96–6.77	0.29–3.70			
**TRIX**						30	*F* = 1.35	0.277
Mean ± SD	4.53 ± 0.16	3.45 ± 0.69	4.17 ± 0.35	3.63 ± 0.60	3.38 ± 0.16			
Median	4.57	4	3.74	3.67	3.32			
Min–Max	3.97–4.97	0.14–4.83	3.50–5.07	2.96–4.51	2.77–4.34			

### Soil characteristics

To determine the carbon storage in the sediments, we used PVC cores 1 m in length (*n* = 62). These cores were inserted by manual hammering. Due to the karst characteristics of the continental shelf, core penetration varied from 0.20 to 1 m. All cores were extrapolated to 1 m long (Howard et al., 2014). Core compaction was less than 5% in all cases, and compaction was not considered for correction in this study due to the coarse sediment composition. In the lab, the cores revealed high heterogeneity over several layers of sediment and were sliced into five cm sections at different intervals (Supplementary material Data base). Slices were selected for LOI, OM% and C_ing_%, C_org_%, TN%, and TP % determination.

### Organic and carbonate content

Each slice (*n* = 298) was weighed before and after drying at 70 °C for 48 h to determine bulk density (BD). Bulk density was calculated as the dry weight of the soil subsamples divided by the volume of the subsample (five cm^−3^) and expressed as g cm^−3^. All samples were homogenized and combusted at 500 °C for 4 h to determine LOI (OM%) and then for 2 h at 900 °C to determine the carbonate content C_ing_ (%) (Kendrick and Lavery, 2001; Heiri et al. 2001). C_org_% (after acidification with 1 N HCl to remove carbonates) and nitrogen content (TN%) were analyzed using a CHN ThermoQuest autoanalyzer (model Flash EA 112, Italy). The C_org_ content (C_org_; g C_org_ cm^3^) of each five cm slice was calculated from the measured C_org_ and the BD of the slice following Eq. (1):

(1) \begin{eqnarray*}{\text{C}}_{org}=z\text{slice*}{\mathrm{BD}}_{\text{slice}}\mathrm{ \ast }{\text{Corg}}_{\text{slice}}/100\end{eqnarray*}

where *z*slice is the slice thickness (cm), and the C_org_% content of the slice is divided by 100 to convert % to grams of C_org_ per gram of dry weight. The amount of carbon stored in each core was calculated by summing the C_org_ content in each depth increment (slice). C_org_ stocks (Mg C ha^−1^) were converted to CO_2equivalents_ by multiplying by 3.67 (conversion factor, ratio of molecular weight CO_2_ to C_org_). Total phosphorus (TP %) was determined by the colorimetric method described by Strickland & Parsons (1972) and Aspila, Agemian & Chau (1976).

### Carbon source

To determine the organic carbon sources in the sediment, isotopic analysis of *δ*^13^C and *δ*^15^N was performed. Surface layers (0–5 cm) of sedimentary cores (*n* = 24) distributed in the north, center, and south of the reserve were selected, considering a water depth gradient of 1 to 5 m with a maximum distance of 25 km from the coast. The subsample for organic carbon analysis was dried, weighed, and then dry-sieved through a one mm mesh to remove coarse inorganic particles. The remaining samples were then acidified with acid 10% (HCl). The residual samples were redried and then capsulated for analyses using a mass spectrometer (Delta V Plus) with an instrumental precision of 0.2%. The Standard material for carbon is Pee Dee Belemnite (PDB) limestone, and the nitrogen standard is atmospheric nitrogen. The *δ*^13^C carbon signal was expressed in parts per thousand (‰), which was obtained by the isotopic ratio of the heavy isotope in relation to the light isotope (Eq. (2)):

(2) \begin{eqnarray*}{\delta }^{13}C(\permil ) \left[ \left( \frac{{R}_{sample}}{{R}_{standar}} -1 \right) \right] \times 1000,R= \frac{{\delta }^{13}C}{{\delta }^{12}C} .\end{eqnarray*}

The relative contribution of different primary producers as potential sources of organic matter in seagrass sediments was estimated using Fits Stable Isotope Mixing Models (SIMMR V. 0. 3) (Parnell et al., 2010; Parnell et al., 2013). We ran the mixing models separately for each water depth (1-5 m) sediment, and we only included as potential sources those primary producers for which both *δ*^13^C and *δ*^15^N values were available: macroalgae blades (*δ*^13^C−13 ± 0.2 ‰, *δ*^15^N 1.74 ± 1.59 ‰), seagrass leaves (*δ*^13^C −11 ± 0.3 ‰, *δ*^15^N 2 ± 0.2 ‰), and mangrove leaves (*δ*^13^C -29 ± 2 ‰, *δ*^15^N 1.1 ± 0.09 ‰) (Duarte et al., 2018; Campbell & Fourqurean, 2009; Vaslet et al., 2015).

### Statistical analysis

The frequency distributions of the water quality characteristics, vegetation structures, and sediments (storage and isotopic signatures) of this study generally did not show normality (based on the normality test of Shapiro–Wilk), so differences in the characteristics of these components between depths were evaluated using the nonparametric Kruskal-Wallis test. Variables that showed normality were analyzed using ANDEVA. These analyses were performed using SigmaPlot 12^®^ software with a significance level of 0.05. Tables 1 and 3 in the Results section show the variables with significant differences according to the depth gradient.

## Results

### Water quality

The trophic state of the water column estimated through the TRIX index showed an average of 3.85 ± 0.12, which is in the oligotrophic range. However, this value varied between oligotrophic and mesotrophic. The LPBR coastal water temperature averaged 29 ± 0.12 °C, while the average water salinity was 35 ± 2.43 ups (Table 1). DO concentrations averaged 6.92 ± 0.16 mg l^−1^, and no hypoxia concentrations were recorded at any depth (Table 1). Light incidence was greatest at 1 m depth, (52 ± 11%), and decreased with depth u<20% at 5 m (Table 1). NO_3_ − + NO_2_ − in the water column of LPBR averaged 5.26 ± 0.79 µmol l^−1^, with the maximum concentration at 3 m depth (Table 1). SRP averaged 0.35 ± 0.02 µmol l^−1^, with the highest values at greater depths (Table 1). NH_4_^+^ averaged 1.20 ± 0.08 µmol l^−1^, with the highest values at the lowest depths. SRSi concentrations in the study area averaged 21.6 ± 2.7 µmol l^−1^, varying from higher to lower concentrations following the depth gradient or coastal distance (Table 1). Chlorophyll-a (Ch-*a*) values averaged 2.59 ± 0.42 µg l^−1^, with the highest concentrations at the lower depths (Table 1).

### Sediment characteristics

Sediments in the LPBR had an average bulk density (BD) of 0.31  ± 0.25 g cm^−3^, and exhibited no significant differences due to the water depth gradient (gl = 4, *H* = 5.69, *P* = 0.223). Concentrations of OM% ranged from 8 to 35%, and these values did show significant differences (gl = 4, *H* = 126.28, *P* < 0.001). C_org_%, ranged from 7 to 15% and decreased with depth (Table 2, gl = 4, *H* = 29.31, *P* < 0.001). C_ing_% increased with water depth and maximun concentration was found at 5 m (gl = 4, *H* = 8.12, *P* = 0.087). TN% and TP% did not show significant spatial variation (gl =49, *F* = 01.81, *P* = 0.142, and gl =49, *F* = 2.10, *P* = 0.097, respectively), while the TN:TP ratio varied between 22 and 42 in the depth gradient (Table 2).

### Seagrass community

**Figure 2:**
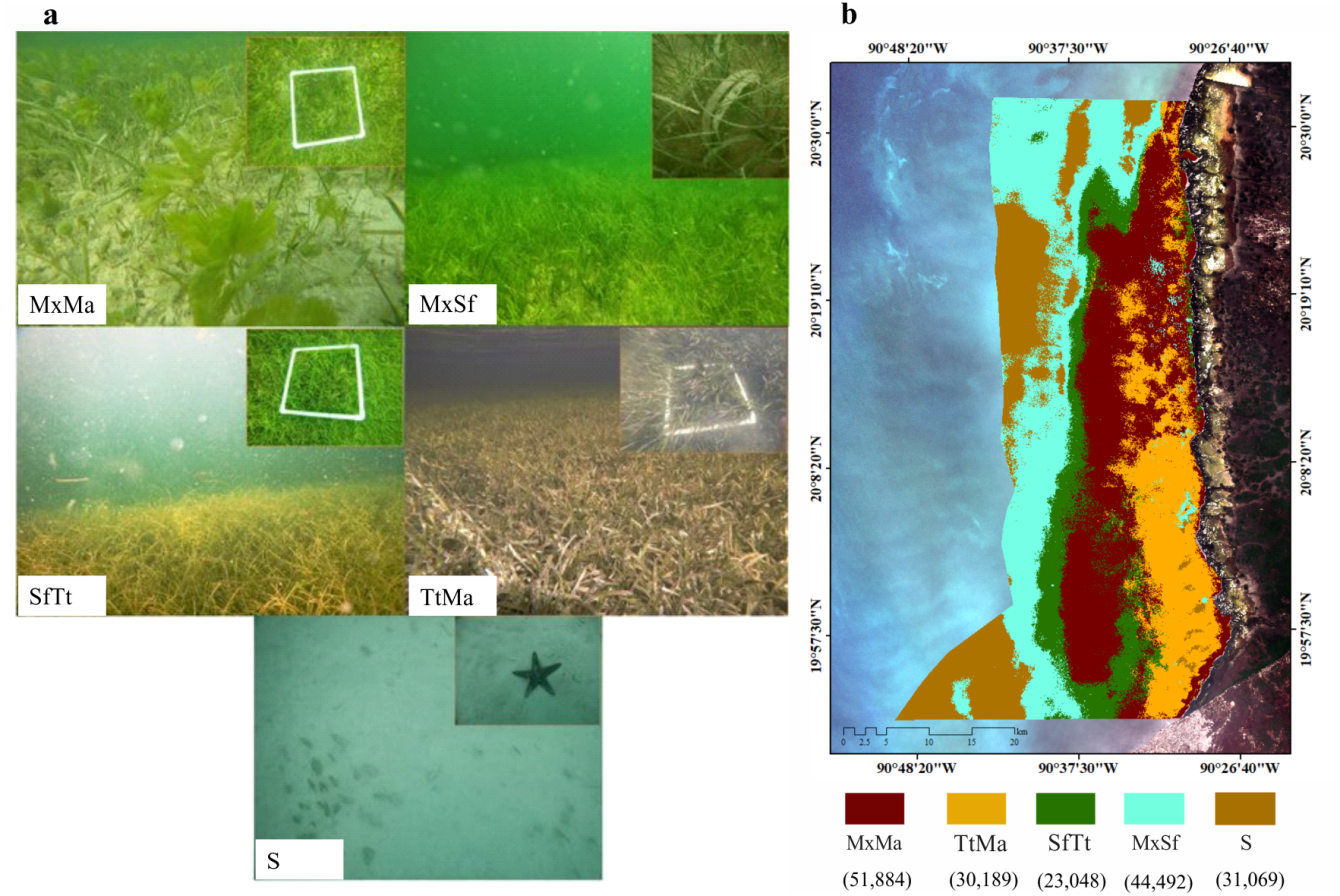
Seagrass meadows. Seagrass distribution. (A) seagrass classes based on species coverage. (B) supervised classification Sentinel 2A, extension of seagrass in LPBR (ha).

Seagrass meadows and macroalgae occupied 82% (149,613 ha) of the LPBR (181, 991 ha). The remainder was substrate without vegetation (31,069 ha) (Fig. 2A). Mixing meadows dominated this area with 51,884 ha of MxMa macroalgae (Fig. 2B). The supervised classification allowed for mapping with 73% accuracy.

Three species, *T. testudinum*, *S. filiforme*, and *H. wrightii*, were recorded in different abundances in both monospecific and mixed meadows. *T. testudinum* dominated*,* with an average coverage of 54 ± 24% and greater coverage at depths of 1 and 2 m. *Syringodium filiforme* showed an average coverage of 45 ± 27%, with the greatest abundance at 3 m depth (50%). *H. wrightii*, showed an average coverage of 27 ± 21%, with the highest value at 3 m (38%). Shoot density of the seagrass species in the LPBR averaged 432 ± 34 shoots m^−2^, registering wide variations between depths. Length of averaged 32 ± 1.33 cm. *T. testudinum* leaves averaged was 33 ± 1.61 cm, with the longest leaves (≈ 80 cm) at a depth of 2 m. At 5 m, *T. testudinum* had the widest leaves (1.2 ± 0.11 cm), and the smallest leaves were found at 1 m (Table 3). Leaf area index (LAI) averaged 4.06 ± 0.44, and the highest average was found at 2 m depth (7.75 ± 1.41). *Syringodium filiforme* showed the longest leaves at 5 m (44 ± 4.67 cm), while *H. wrightii* had the smallest average leaves (15.6 ± 2 cm). In relation to biomass, seagrass meadows in the LPBR averaged 119 ± 13 and 510 ± 46 g Dw m^−2^ in aboveground and belowground biomass, respectively, with the greatest values measured at 2 m depth (aboveground at 196 ± 42 g Dw m^−2^, and belowground at 768 ± 157 g Dw m^−2^). *Thalassia testudinum* had the largest contribution (79%) to total biomass, which decreased at greater depths when replaced by *S. filiforme* (Tabla 3). The above/below biomass ratio (AB:BW) averaged 0.32, which corresponded to 81% of the total biomass (Table 3). Macroalgae coverage decreased with water depth, and there were no significant differences between water depths (Table 4, gl =4, *H* = 10.51, *P* = 0.033).

**Table 2:** Summary of sediment quality parameter, organic matter (OM %), organic carbon (Corg %), inorganic carbon (Cing %), total nitrogen (TN%), total phosphorus TP (%), ratio nitrogen/phosphorus (N/P) measured in LPBR at different water depths(1–5 m depth), showing mean ± SD.

Depth (m)	Bulk density (g cm^−3^)	OM (%)	C_org_ (%)	C_ing_ (%)	TN (%)	TP (%)	N/P
1	0.28 ± 0.22	35.66 ± 20.62	17.28 ± 6.70	3.79 ± 3.32	2.72 ± 1.58	0.18 ± 0.07	41.60 ± 34.45
2	0.29 ± 0.20	22.38 ± 13.88	11.10 ± 3.79	3.67 ± 1.07	2.41 ± 0.79	0.25 ± 0.08	22.06 ± 5.70
3	0.40 ± 0.29	11.06 ± 5.29	8.64 ± 0.88	3.82 ± 0.79	2.10 ± 1.18	0.20 ± 0.06	27.17 ± 17.36
4	0.30 ± 0.24	12.27 ± 7.51	8.33 ± 1.19	3.33 ± 0.53	3.17 ± 0.97	0.22 ± 0.04	32.07 ± 11.95
5	0.30 ± 0.2	8.48 ± 5.48	7.38 ± 1.53	3.82 ± 0.74	3.78 ± 2.17	0.20 ± 0.01	42.07 ± 26.26

**Table 3:** Summary of seagrass community. Summary of seagrass community and structure measured in LPBR at different water depths (1–5 m depth), showing mean ± SD, median, minimum and maximum values. Statistical data test (ANDEVA and Kruskall-wallis test). Bold represent statistical differences.

**Species**	**Depth**	**km**	**AB gDW m** ^−2^	**BW gDWm** ^−2^	**Ratio AB:BW**	**Leaf length (cm)**	**Leaf width (cm)**	**#Leaf/** **shoot**	**#shoots m** ^−2^	**Coverage (%)**	**LAI**
***T. testudinum***	**1**	0.01–8									
Mean ± SD			95 ± 49	768.35 ± 443	0.12	23 ± 9	0.8 ± 0.16	4 ± 1	460 ± 400	75 ± 25	3.14 ± 2.2
Median			103.23	871.32		20.75	0.82	4	270	80	2.27
Min–Max			17.64–158.62	29.41–1392		15–44	0.50–1	3–6	168–1235	40–100	0.67–6
***T. testudinum***	**2**	2–10									
Mean ± SD			196 ± 163	758.22 ± 493	0.25	42 ± 20	0.9 ± 0.20	4 ± 0.7	434 ± 230	74 ± 31	7.75 ± 5.45
Median			169.7	598.23		35.62	0.8	4	416	90	7.9
Min–Max			9.4–560	70.29–1647.05		15.25–80.2	0.6–1.35	3–6	112–840	20–100	0.5–15
***S. filiforme***											
Mean ±SD			45 ± 10	466 ± 432	0.09	19 ± 16			794 ± 331	45 ± 45	
Median			46	250		18			794	30	
Min–Max			35–45	185–964		13–26.5			560–1029	30–75	
***T. testudinum***	**3**	3–14									
Mean ±SD			77 ± 68	438.52 ± 302	0.17	29 ± 8	0.9 ± 0.23	4 ± 1	334 ± 169	46 ± 28	3.44 ± 2.43
Median			48.97	447.05		27	0.8	4	294	40	2.45
Min–Max			10–279.41	21–1050		15.77–49.67	0.3–1.5	2–6	58–794	10–100	0.23–8.76
***S. filiforme***											
Mean ±SD			40 ± 38	125 ± 137	0.32	30 ± 11			364 ± 294	50 ± 33	
Median			25.98	89.4		29			121.5	30	
Min–Max			3.52–129.41	7.05–435.29		11.5–53			117–1058	15–100	
***H.wrightii***											
Mean ± SD			13 ± 7	79 ± 35	0.17	20 ± 7			574 ± 234	38 ± 33	
Median			13.13	94.11		21			587	30	
Min–Max			5.88–23.52	17.64–102.94		10–28			300–823	10–75	
***T. testudinum***	**4**	10–17									
Mean ±SD			53 ± 46	201 ± 167	0.26	34 ± 12	1 ± 0.19	4 ± 0.8	345 ± 282	53 ± 27	3.84 ± 4.12
Median			35.88	182.35		27	0.9	3.5	264	50	2.66
Min–Max			13.52–153.52	14.11–568.82		21.65–56.50	0.8–1.52	2–5	25–882	15–100	0.20–12.37
***S. filiforme***											
Mean ±SD			121 ± 132	250 ± 166	0.48	29 ± 12			772 ± 646	41 ± 28	
Median			52.94	238.52		24			735	37.5	
Min–Max			5.88–411.76	29.41–517.64		12–14			64–2000	10–90	
***H.wrightii***											
Mean ± SE			171 ± 138	346 ± 266	0.49	13 ± 1			1418 ± 252	25 ± 14	
Median			175	344		13			1239	25	
Min–Max			5.88–329	37–658		12–14			1239–1596	15–35	
***T. testudinum***	**5**	11–25									
Mean ± SD			64 ± 60	113 ± 51	0.56	35 ± 15	1.2 ± 0.3	4 ± 1	128 ± 80	38 ± 11	1.3 ± 0.8
Median			48.52	126.47		38.77	1	3	128	40	1.73
Min-Max			11.76–147.35	41.17–161.76		16.45–60	0.6–1.8	2–5	10–252	15–50	0.1–2.17
***S. filiforme***											
Mean ±SD			78 ± 60	154 ± 141	0.50	44 ± 18			573 ± 329	48 ± 20	
Median			64.7	100.88		44.5			646	50	
Min–Max			11.47–191.17	52.94–511.76		14–64			144–1000	10–80	
***H.wrightii (n* = 1*)***											
Mean ± SD			5.8	170.58	0.03	14 ± 1.5			466 ± 274	27 ± 20	
Median						14			411	20	
Min–Max	*** ***					13–16			224–764	10–80	
***T. testudinum***	**P**					**0.011**	**0.003**	0.066	**0.007**	**<0.001**	**0.003**
	**Critical value**					***F*= 13**	***F*= 16**	*F* = 2.31	***F*= 3.84**	***F*= 5.46**	***H*= 16.39**
	**gl**					**4**	**4**	74	**74**	**79**	**4**
***S. filiforme***	**P**					**0.013**			0.326	0.811	
	**Critical value**					***F*= 4.07**			*H* = 3	*H* = 0.96	
	**gl**					**47**			3	3	
***H.wrightii***	**P**					0.322			**0.013**	0.570	
	**critical value**					*F* = 1.37			***F*= 9.89**	*F* = 0.62	
	**gl**					8			**8**	7	

**Table 4:** Macroalgae coverage. Summary of macroalgae coverage (%) measured in LPBR at different water depths (1–5 m depth), showing mean ± SD, median, minimum and maximum values.

Depth (m)	Macroalgae coverage (%)	Min	Max	Median
1	39 ± 16	20	55	40
2	29 ± 16	10	50	30
3	16 ± 10	5	40	10
4	15 ± 6	10	20	15
5	27 ± 18	15	40	27.5

### Seagrass carbon stocks and sources

**Figure 3:**
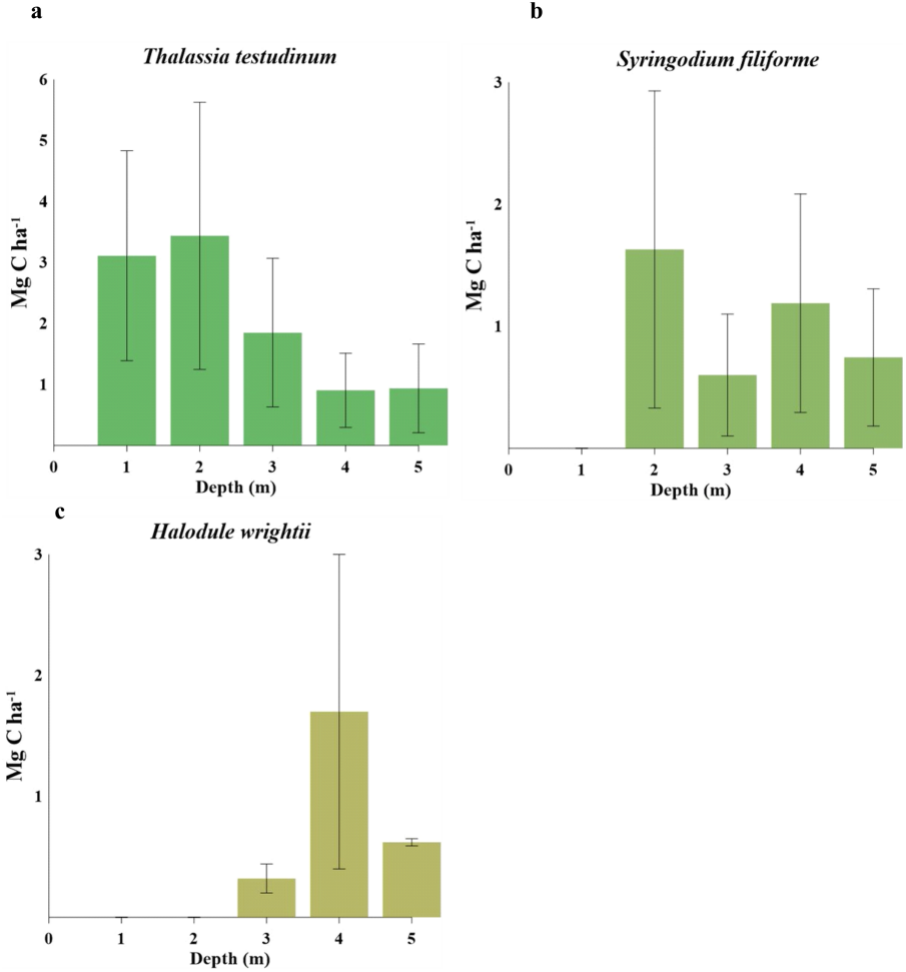
Carbon stocks of seagrass species at different water depths(1–5 m depth): (A) comparison of *Thalassia testudinum*. (B) comparison of *Syringodium filiforme*. (C) comparison of *Halodule wrightii*.

**Figure 4:**
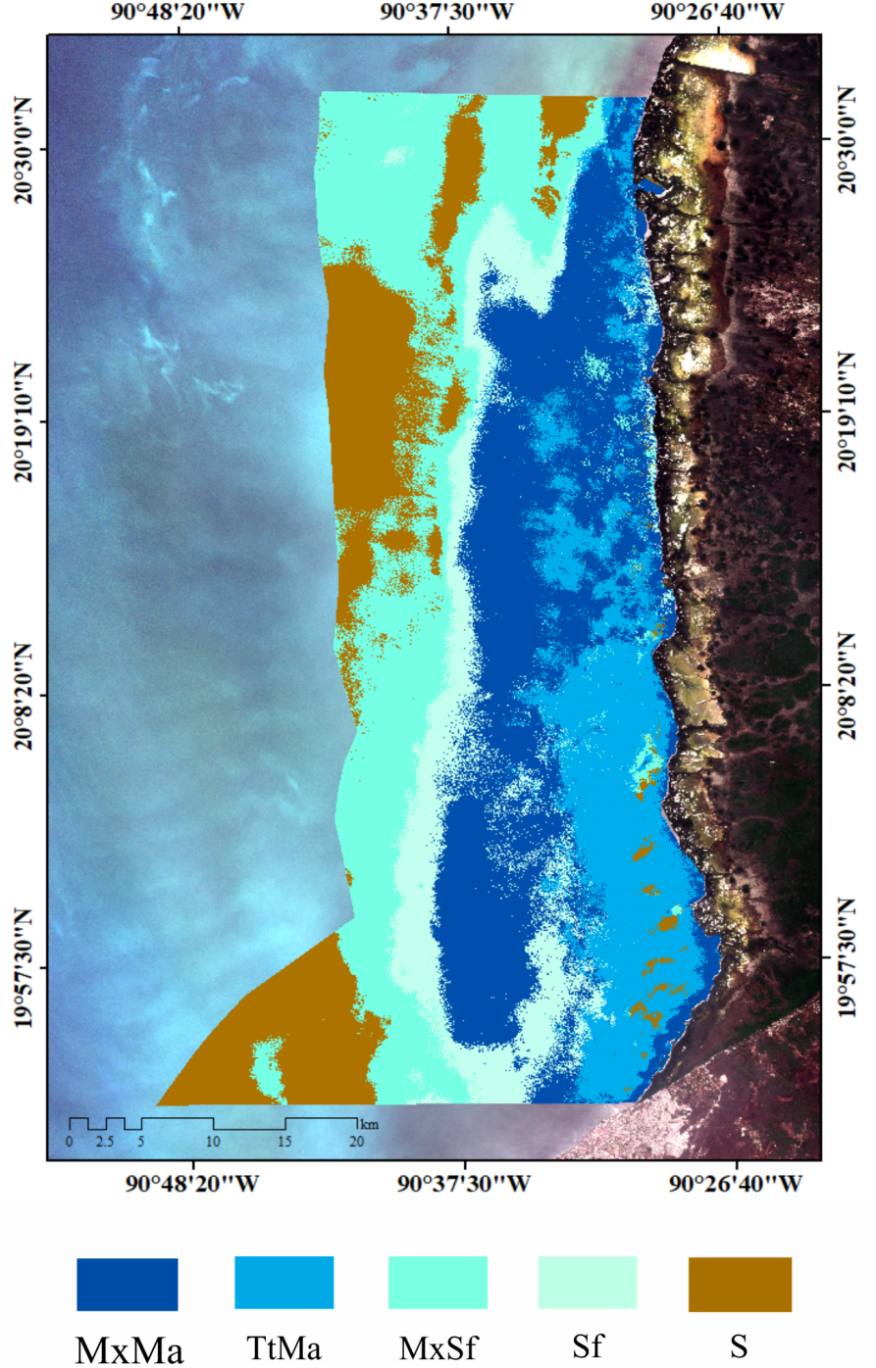
Carbon stocks (biomass + sediments (top 1 m)) of seagrass classes in LPBR.

Biomass organic carbon averaged 2.2 ± 1.7 Mg C ha^−1^, with statistically significant differences between water depths (gl =4, *H* = 13.49, *P* = 0.009, Table 5). *T. testudinum* averaged 2 ± 1.7 Mg C ha^−1^ and decreased with water depth (Fig. 3A). *S. filiforme* and *H. wrightii* averaged 0.88 ± 0.78 and 0.89 ± 1 Mg C ha^−1^, respectively, and did not showed a trend in the gradient (Fig. 3 b,c). Statistical differences between species were found (gl =4, *H* = 22.44, P = <0.001). In sediment (C_sed_), C_org_ storage net averaged 131 ± 118 Mg C ha^−1^ (gl =4, *H* = 13.77, *P* = 0.008). In the top 1 m, this stock was 318 ±  215 Mg C ha^−1^, with a maximum of 463 ± 267 Mg C ha^−1^ at 1 m water (gl = 4, *H* = 6.02, *P* = 0.197) (Table 5). The C _ing_ stock averaged 133 ± 104 Mg C _ing_ ha^−1^, varying with respect to depth (gl =4, *H* = 0.64, *P* = 0.958; Table 5). The seagrasses ecosystem carbon stock (C_bio_ + C_sed_) in Los Petenes Biosphere Reserve was 47Tg C (Table 6). Considering the extension of each type of meadow, those in the MxMa stored the largest C_org_ of the study area (34%). The seagrass meadows dominated by SfTt represented the smallest stock in the area, with 8% of the total (Fig. 4).

**Table 5:** Carbon stores in aerial and belowground biomass. Carbon stores in aerial and belowground biomass. Net organic and inorganic carbon storage and at 1 m in sediment, showing mean ± SD.

Depth (m)	Aboveground and belowground Mg C ha^−1^	Sediment Mg C ha^−1^ Net	Sediment Mg C ha^−1^ Top 1 m	Sediment Mg Cing ha^−1^ Top 1 m
1	3.1 ± 1.7	249 ± 183	463 ± 267	131 ± 119
2	3.7 ± 2.3	147 ± 137	329 ± 225	130 ± 103
3	1.8 ± 1.1	103 ± 50	301 ± 184	135 ± 85
4	1.9 ± 1.3	96 ± 76	271 ± 230	129 ± 122
5	1.1 ± 0.9	77 ± 42	229 ± 207	125 ± 122

The surface of seagrass sediments averaged *δ*^13^C of −17 ± 3 ‰ and *δ*^15^N of 3 ± 1 ‰. In shallow areas (1–2 m), the *δ*^13^C varied between −10 and −24 ‰; in the deeper areas (4–5 m), *δ*^13^C varied between −12 and −17 ‰, with statistically significant differences between water depths (gl = 23, *F* = 3.37, *P* = 0.030, Table 7).

**Figure 5:**
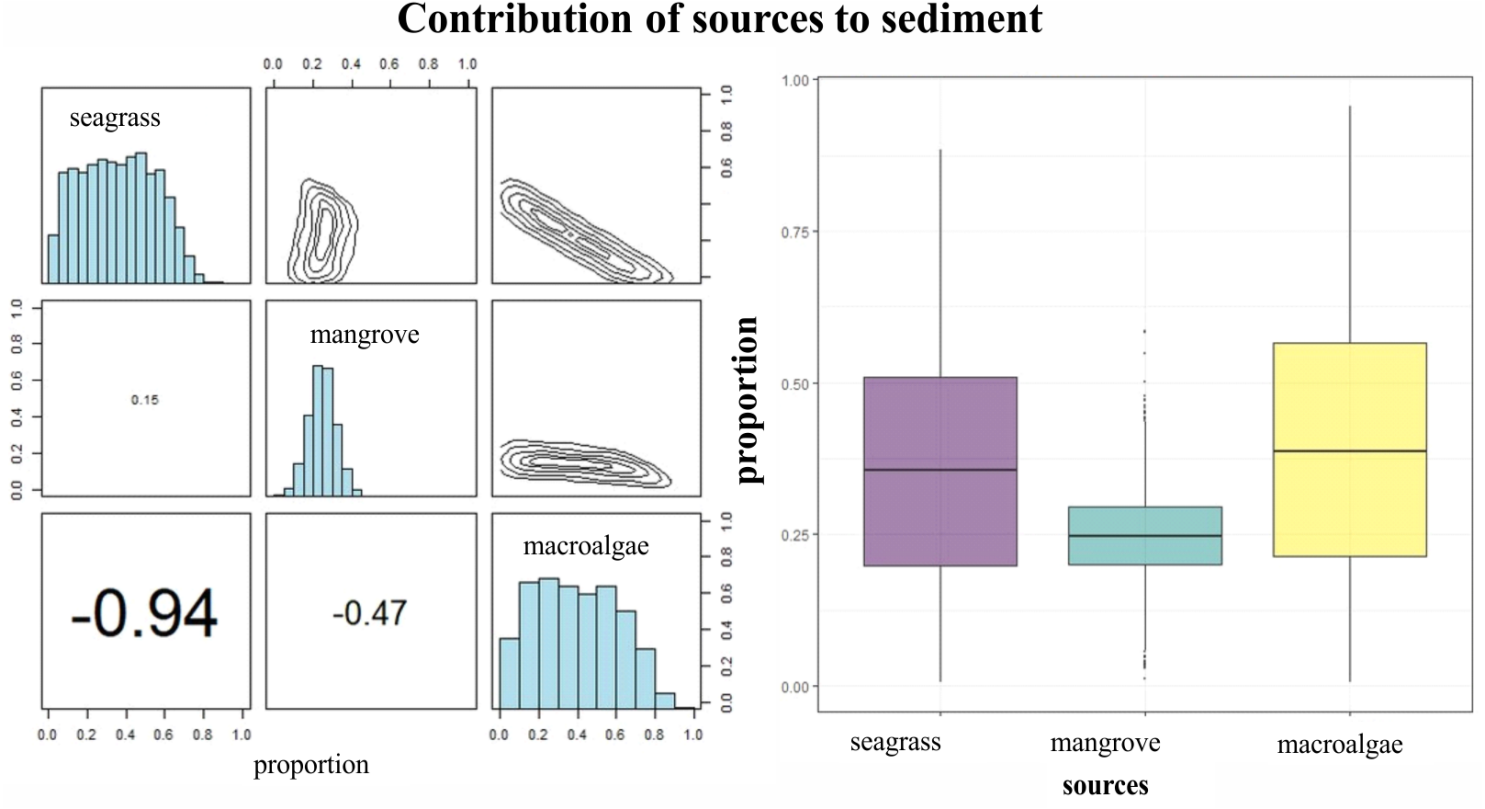
Contribution of seagrass, macroalgae and mangrove to the accumulated carbon organic in seagrass (A, B) sediments in the LPBR calculated using Bayesian mixing models. Matrix plots for seagrass (A) sediments, showing probability distributions of each source (in diagonal panels), the joint probability between pairs of sources (in top panels), and the correlations between pairs of sources (in bottom panels). (B) Box plots of the proportional contribution by each source to seagrass sediments. Boxes extend from the 25th to 75th percentiles and lines inside boxes represent mean values.

*δ*^15^N of LPBR sediment ranged from 2 to 3%, and there were no significant differences in the depth gradient (gl = 23, *F* = 0.64 *P* = 0.637; Table 6). Mean *δ*^13^C and *δ*^15^N values of seagrass sediments were within the region defined by *δ*^13^C and *δ*^15^N mean values of primary producers (Fig. 5A). The SMMIR mixing models identified sources of organic matter in seagrass sediments (Fig. 5B and Table 7). Seagrass leaves and macroalgae blades were the major potential contributors in seagrass sediments (mean ± SD proportion = 0.39 ± 0.19 and 0.36 ± 0.21, respectively), while mangrove leaves had a minor contribution (0.24 ± 0.21). The seagrass contribution increased with water depth (Table 7).

**Table 6:** Carbon stoks by seagrass classes, total biomass and sediment top 1 m, showing mean ± SD. Ecosytem carbon stock and CO2 euivalences in LPBR.

Class	Extension (ha)	Biomass Mg C ha^−1^	Sediment Mg C ha^−1^ top 1 m	Ecosystem carbon stock Tg C	Ecosystem carbon stock TgCO_2eq_
MxMa	51,884	2.7 ± 1.90	307 ± 185	16	59
MxSf	44,492	0.82 ± 0.60	315 ± 299	14	51
TtMa	30,189	2.66 ± 1.55	411 ± 226	13	48
SfTt	23,048	1.76 ± 0.98	155 ± 98	4	15
**TOTAL**	**149,613**		** **	**47**	**173**

**Table 7:** *δ*^13^C and *δ*^15^N values of seagrass sediments. Mean ± SD *δ*^13^C and *δ*^15^N values of seagrass sediments, proportional contribution by each source to seagrass in LPBR at different water depths (1–5 m water depth).

**Depth** **(m)**	*δ* ^**13**^ **C**	*δ* ^**15**^ **N**	**BD** **(g cm** ^−3^ **)**	**OM** **(%)**	**Seagrass**	**Macroalgae**	**Mangrove**
1							
Mean ± SD	−21 ± 4.2	3 ± 2	0.2 ± 0.2	26 ± 9	0.50 ± 0.25	0.38 ± 0.25	0.10 ± 0.07
Median	−21	4	0.23	25.94			
Min–Max	−24–14	1–6	0.12–0.6	12–36			
2							
Mean ± SD	−17 ± 3.7	4 ± 0.6	0.2 ± 0.1	35 ± 19	0.53 ± 0.26	0.37 ± 0.26	0.09 ± 0.06
Median	−19	4	0.25	31			
Min–Max	−19–11	3–5	0.04–0.3	17–57			
3							
Mean ± SD	−16 ± 1.9	3 ± 1	0.5 ± 0.2	15 ± 5	0.59 ± 0.26	0.32 ± 0.25	0.07 ± 0.05
Median	−16	4	0.5	14			
Min–Max	−18–13	1–4	0.3–0.8	10–22			
4							
Mean ± SD	−16 ± 0.4	3 ± 0.6	0.2 ± 0.07	12 ± 5	0.59 ± 0.26	0.33 ± 0.07	0.07 ± 0.05
Median	−16	3	0.2	9			
Min–Max	−17–16	3–4	0.08–0.2	7–17			
5							
Mean ± SD	−14 ± 1	3 ± 2	0.3 ± 0.1	10 ± 9	0.62 ± 0.25	0.30 ± 0.25	0.06 ± 0.04
Median	−14	3	0.3	7			
Min–Max	−15–13	0.2–4	0.16–0.4	5–28			

## Discussion

### Water quality and sediment characteristics

The marine area Los Petenes Biosphere Reserve showed general oligotrophic conditions. This area, which is on a karstic geomorphological coast, receives groundwater discharge from springs and runoff from channels that connect mangrove areas with the sea (Agraz-Hernández et al., 2012). Exchange of nutrients at the site reflects high concentrations of OM%, C_org_%, and TN% in the areas near the coastline (1–2 m water depth). The concentration of these nutrients in the sediment is associated with the type of organic matter that they receive from both mangroves and submerged aquatic vegetation (SAV), as well as degradation patterns, residence times of the accumulated matter, and the chemical balance of carbonate (Koch, 2001). Carbonate concentrations exceeded 20% in the deep zone (Table 2). N/P ratios show that sediments in the reserve are marine type and are influenced by terrigenous material (Hernández, 2000). Isotopic mangrove signatures in the superficial sediments at 1 m depth support this finding (Fig. 5; Table 7). In general, concentrations of registered nutrients indicate that there is no limitation of primary production, at least in the first meters of water depth, since the concentrations are in the tolerance range for seagrasses. In karst systems, it is common to observe P limitations (Álvarez Góngora & Herrera-Silveira, 2006); the connectivity between mangroves and seagrasses in this area, provides favorable conditions for the development of quality SAV. However, this leads to significant vulnerability if groundwater discharges begin to show signs of pollution, as they have in the northern and eastern regions of the Yucatan Peninsula (Herrera-Silveira & Morales-Ojeda, 2009; Arcega-Cabrera, Barroso & Oceguera-Vargas, 2014; Kantun-Manzano et al., 2018). However, due to the connectivity between mangroves and seagrasses, the light and concentration of nutrients in this area favor conditions for the healthy development of SAV.

### Seagrass complexity

The large area covered by SAV indicates good conditions, as reflected in the spatial continuity of the seagrass meadows, morphometric characteristics, and their biomass (Table 3). These characteristics of LPBR seagrasses are useful as baseline data for monitoring the health of the ecosystem. Rapid coastal development experienced by these areas may allow for the identification of responses to different environmental stressors (Tomasko & Lapointe, 1991; Lirman & Cropper, 2003; Lee, Park & Kim, 2007). There was also spatial variability in the specific morphometric and structural characteristics of the seagrass community in the study area with respect to the depth gradient and distance from the coast. This implies changes in variables such as salinity and transparency (Table 1), which control seagrass productivity (Fourqurean, Zieman & Powell, 1992).

The structural variability of seagrass beds with respect to depth gradient (Table 3) indicates an area where *T. testudinum* dominates at lower depths, with the presence of patches of *H. wrightii* (coverage of <5%); at greater depths, the dominant species was *S. filiforme*, which is consistent with tropical locations (Tribble, 1981). In the LPBR, *T. testudinum* and *H. wrightii* dominated areas with greater salinity variability; *S. filiforme* was common in areas of less salinity variation and lower light requirements, which encouraged its dominance in deeper areas (Zieman, Fourqurean & Iverson, 1989; Hall et al., 1999; Lirman & Cropper, 2003). Biomass, coverage, density, and LAI of *T. testudinum* was lowest in the deepest zone (5 m) (Table 3) due to the lowest incidence of light (Olesen et al., 2002; Enríquez & Pantoja-Reyes, 2005). Additionally, the primary roots of *T. testudinum* were observed to be approximately 30 cm long. Greater belowground biomass and rhizomal elongation determine the depth of anchorage of the species and allow it to tolerate erosion processes, therefore contributing to the C _org_ stock. In contrast, the dominant seagrass at 5 m was *S. filiforme*, suggesting that the light requirements vary between species due to the physiological characteristics and morphological adaptations of each (Lee, Park & Kim, 2007).

Environmental conditions in the LPBR favor the growth of seagrasses (Table 3). Leaf lengths were greater than lengths reported for other regions of the Gulf of Mexico and the Caribbean (Hackney & Durako, 2004; Arellano-Méndez et al., 2014; Gallegos-Martínez et al., 1993; van Tussenbroek, 1998). Additionally, the total biomass (above + belowground = 863  ± 478 g Dw m^−2^) was higher than the reported global scale value of ∼461 g Dw m^−2^ (Duarte & Chiscano, 1999) and lower than estuarine zone estimates (879 g Dw m^−2^). The structural complexity and favorable environmental conditions in the LPBR reflected in the quantity and quality of the ecosystem services these seagrasses provide, such as storing carbon in high concentrations (Fig. 2; Table 6).

### Carbon stocks and sources along a depth gradient

In the study area, carbon stored as biomass in seagrasses showed a lower average (2.2 ± 1.17 Mg C ha^−1^) than those reported on a global scale (3 ± 0.4 Mg C ha^−1^), but they were greater than values from the South Atlantic meadows (1 ± 0.5 Mg C ha^−1^; (Fourqurean et al., 2012a). Mean sediment stock at the top 1 m (318 ± 215 Mg C ha^−1^) was in the range reported for seagrasses in the tropical region of Australia (268 Mg C ha^−1^) and was greater than the global estimates (194 ± 20 Mg C ha^−1^) (Fourqurean et al., 2012a). These results suggest that the study area has a high capacity to capture and store coastal carbon, making it highly relevant for the mitigation of greenhouse gas emissions; it must therefore remain protected.

Both aerial and underground carbon storage showed spatial variability related to depth/distance to the shoreline (Table 5). Some studies for other species have indicated that C_org_ storage and sequestration rates in seagrass sediments are higher in shallow meadows and at moderate salinities (Mateo & Romero, 1997; Serrano et al., 2014). The increase in depth implies a reduction in irradiance and a decrease in salinity, which in part affect the productivity of the seagrasses, their structural complexity, and the species composition, with ultimate consequences on carbon stocks. Hydrodynamics influences the structural complexity of seagrasses and probably influences the storage and carbon fluxes in seagrasses (Mateo & Romero, 1997; Koch et al., 2006; Serrano et al., 2014; Dahl et al., 2016). Although this variable was not evaluated in this study, currents in this area is the lowest (1 m sec^−1^) of the three coasts of the Yucatan Peninsula (López & Sierra, 1998), Furthermore, in combination with the low tidal range (<1 m), hydrodynamic energy must be low, favoring processes such as sedimentation, retention, and decomposition of materials produced both locally and regionally. Such is the case for the area near the coast that receives contributions of organic matter from the adjacent mangrove forest (Fig. 5).

Isotopic values of *δ*^13^C from surface sediments varied between −10 and −24 ‰ in relation to distance to the coast. This indicates different sources of organic carbon in the seagrass soils of this area. Near the coast and up to 7 km from the coast, the isotopic signatures averaged *δ*^13^C of −21 ± 4.2 ‰ (Table 7), was is similar to the mangrove sediments enriches with carbonates (Garcias-Bonet et al., 2019). Mangrove contribution to carbon storage decreased with depth, the inverse pattern of the contribution of seagrass is observed (Table 7). Macroalgae blades and seagrass leaves in seagrass sediments were strongly negatively correlated (−0.94) (Fig. 5A). This indicates the model could not determine the principal carbon source in sediment, indicating that if macroalgae blades contributed to seagrass sediments at the top of their outcome probability range, seagrass leaves most likely contributed at the bottom of their probability range (Fig. 5A). Stocks of organic carbon in sediments of seagrasses of this protected area were 24% allochthone sources (mangroves), while 76% were of autochthon origin (macroalgae and seagrass) (Fig. 5B). Therefore, the identification of potential sources and contributions to sediments based on stable isotopes needs to be interpreted with care. These results confirm, first, the connectivity between two coastal ecosystems (mangroves and seagrasses) (De Boer, 2000; Serrano et al., 2014) and, simultaneously, the role of seagrasses as sediment traps and sequestrants of allochthonous carbon (Mellors et al., 2002; Hendriks et al., 2008; Samper-Villarreal et al., 2016).

### Conservation implications

Carbon stocks in seagrass meadows of this protected natural area vary according to the extent of each type of seagrass meadow, with a total of 47 Tg C_org_ (Table 6). This value contrasts with the estimate of Thorhaug et al. (2017) of 37–38 Tg C_org_ for the entire Gulf of Mexico estimates for Mexico (48 Tg C_org_), where the higher stock is located in the Yucatan Peninsula (Herrera-Silveira et al., 2020). Seagrasses, mangroves, and salt marshes, are collectively called blue carbon ecosystems (Howard et al., 2014; Howard et al., 2017). Disturbance of these system can increase CO_2_ emissions as the carbon in the necromass and surface sediment oxidizes. Mineralization of the carbon stored in LPBR could relase 173 Tg of CO_2eq_. This corresponds to emissions generated by 27% of the current Mexican population, based on per capita emissions from fossil fuel consumption estimated for 2009 (3.72 t CO_2_) (Cavazos et al., 2013). The loss of vegetation cover in the Gulf of Mexico has been progressively decreasing, with an estimated 50% decrease from 1,927,500 ha in 1992 to 947,327 ha in 2017 (Duke & Kruczynski, 1992; Thorhaug et al., 2017); this loss is equivalent to an estimated annual loss rate of vegetation in the region of approximately 3% over a span of 25 years. The seagrass meadows in the LPBR have the largest extension of seagrasses in Mexico, with 149,613 ha currently reported. Therefore, if the coastal vegetation in this reserve disappeared at the same estimated annual rate, in 25 years, only 30% of the current extension would remain. This would likely significantly reduce the ability of the LPBR to offer its current ecosystem services.

The coastal platform of the Yucatan Peninsula is shallow with a steep slope of ∼1:1000 (Zavala-Hidalgo et al., 2003); the bottom in areas near the coast, therefore, are covered with submerged aquatic vegetation, mainly seagrasses dominated by *T. testudinum* (Espinoza-Avalos, 1996; van Tussenbroek et al., 2014). Hydrodynamic conditions, such as the speed of the currents and their exposure to hydrometeorological events, such as hurricanes, storms, and cold fronts, could explain the differences in the seagrass cover of the three coasts of the Yucatan Peninsula (Day et al., 2019). On the coast of Quintana Roo, currents average 25 cm sec^−1^, and patches of scattered seagrasses covering between 10 and 50% of the available area (Badan Jr et al., 2005; Arellano-Méndez, Morales-Ojeda & Herrera-Silveira, 2014). On the northern Yucatan coast, currents range between 10 and 20 cm sec^−1^, with seagrass patches covering between 40 and 80% of the available area (Appendini et al., 2012; Kantun-Manzano et al., 2018). Finally, on the coast of Campeche, where the Los Petenes protected area is located, currents are very low at <10 cm sec^−1^ (López & Sierra, 1998), favoring the extension and coverage of seagrasses.

## Conclusions

The LPBR has the largest extension of the seagrass community in the Gulf of Mexico. Carbon stored (76%) is from seagrass and macroalgae sources. Allochthon contribution decreased and seagrass contribution increased with depth. The results of this study improve estimations of organic carbon storage (47 Tg) in a marine protected area and demonstrate the importance of blue carbon stocks and connectivity between mangrove and seagrass ecosystems in the subtropics.

## Supplemental Information

10.7717/peerj.12109/supp-1 Supplemental Information 1 Meadows descriptions

10.7717/peerj.12109/supp-2 Supplemental Information 2 Summary of seagrass community and structure measured in LPBR at different classes Summary of seagrass community and structure measured in LPBR at different classes, showing mean ± SD, median, minimum and maximum values.Statistical data test (ANDEVA and Kruskall-wallis test). black letters represent statistical differences.

10.7717/peerj.12109/supp-3 Supplemental Information 3 Seagrass, water and sediment data These data were used for statistical analysis to compare depths.

## Additional Information and Declarations

### Field Study Permissions

The following information was supplied relating to field study approvals (i.e., approving body and any reference numbers): Field experiments were approved by National Commission of Natural Protected Areas (CONANP).

### Data Availability

The following information was supplied regarding data availability: Raw measurements are available in Supplemental Files.
