# Supplementary material for: Seagrass contribution to blue carbon in a shallow karstic coastal area of the Gulf of Mexico
Source: PeerJ. 2021 Sep 10;9:e12109. doi: 10.7717/peerj.12109 (PMC8436957; doi:10.7717/peerj.12109)
Supplement: Supplemental Information 1 [file peerj-09-12109-s001.docx]

Supplementary Material

# Description of community seagrass by meadows type.

For supervised classification (Fig x) We found five class of seagrass meadows consider their structure (coverage (%) and observational cases). We don’t considerer the density value because small species of seagrass (ej. *H. wrigthii*) tend to have higher density than medium and large species *(T. testudinum* and *S. filiforme)* (Kruczynski and Fletcher 2012). The class are: monoespecific meadows of *Thalassia testudinum* with macroalgae (TtMa), Mixing seagrass meadows (*T. testudinum, S. filiforme, H. wrightii*) with macroalgae (MxMa), Mixing meadows dominated by *Syringodium fliforme* (MxSf), *Syringodium filiforme* and *Thalassia testudinum* beds (Sf Tt) in this class just a one sample with *H. wrigthii* was found (Table 2), finally only with observations we defined the sand class (S) that correspond to substrate with low (<5 % coverage) and inexistent seagrass beds n=57.

**Characteristics of seagrass meadows and carbon distribution**

## Coverage

The dominant species was *T. testudinum* with an average coverage of 50 ± 27.18%, being higher in the Tt and MxMa grasslands (Table 2), decreasing significantly in the area dominated by *S. filiforme* (P <0.046). Regarding *S. filiforme*, it presented an average coverage of 46 ± 24.89%, with the highest abundance (50%) in MxMa meadows, followed by monospecific seagrass of this species with 49 ± 27%, its variability in coverage was significantly different (Table 2; P = 0.027). For the H. wrightii species, the average coverage was 27 ± 21.37%, with the highest value in the MxSf seagrass meadows (38%), however, it did not present significant differences (P = 0.18) between the seagrass medowss. The coverage between the species presents significant differences (P = 0.002).

## Density

The density of seagrass shoots in the LPBR presented an average of 432 ± 34 m2 shoots. By species it was observed that *T. testudinum* on average has 336 ± 244 m^2^ shoots, with the highest density in the MxMa and Tt meadows with 400 ± 293 and 388 ± 186 m^2^ beams respectively (Table 2), these variations are statistically different (P = 0.006). The beam density of *S. filiforme* presented an average of 590 ± 435 m^2^ shoots, presenting its highest density in the MxMa and Sf seagrass area, the differences between grasslands in the shoot density (Table 8) did not vary significantly (P = 0.223). Regarding *H. wrightii* the average density is 725 ± 451 m^2^ shoots, registering wide variations, being the seagrass dominated by *S. filiforme* where the highest densities are found, however, there are no significant differences (P = 0.336). Between species, the density values were statistically different (P = 0.003).

## Biomass

The seagrass meadows in the LPBR presented an average biomass in avobe and belowground tissue of 119 ± 13 and 510 ± 46 g Dw m^-2^, respectively. MxMa seagrass presented the highest total biomass with 764 ± 542 g Dw m^-2^ followed by Tt meadows (730 ± 436 g Dw m^-2^) (Table 2). In general T. testudinum has an above and belowground biomass of 69 ± 135 and 105 ± 242 g Dw m-2 where the highest average value was recorded in its Tt meadows (730 ± 436) with significant differences between the type of meadow (Table 2; P = 0.001). For its part, *S. filiforme* has an above biomass of 63 ± 82 g Dw m^-2^ and underground of 169 ± 198 g Dw m^-2^ without significant differences between seagrass meadows (P = 0.166). For *H. wrightii* the average values of above and below ground biomass were 46 ± 100 and 141 ± 188 g Dw m^-2^. This species has no significant differences between the type of meadow (P = 0.729).

## Morphometry

The length of the leaf in the reserve presents a general average of 32.48 ± 1.33 cm. For *T. testudinum* the average leaf length was 33 ± 1.61 cm (Table 2). MxMa meadows have the longest leaves, without significant differences (P = 0.325). The highest average width of the leaves occurs in MxSf meadows (1 ± 0.2 cm) and the smallest in Tt meadows (0.8 ± 0.2). The highest number of leaves per beam for this species was recorded in Tt and MxMa meadows with an average value of 4 ± 1 and 4 ± 2 respectively. Regarding the Leaf Area Index (LAI), the reserve has an average value of 4.06 ± 0.44, being the Tt monospecific meadow where a higher average is presented (5 ± 4), these differences are significant (P = 0.043).

In the case of *S. filiforme* the average length of the leaves was 33.48 ± 14.6 cm. The leaves of greater length appeared in the seagrass Sf (36 ± 15.4 cm). Among seagrass types, there are no significant differences (P=0.827). *Halodule wrightii* presents the smallest leaves with an average value of 15.6 ± 2 cm, where in the Sf meadows it presents its longest leaves, however, there are no significant differences (P = 0.204).

References

Kruczynski, W.L. and P.J.Fletcher (eds).2012.Tropical Connections: South Floridas´s marine eviroment.IAN.Press,University of Maryland Center for Enviroments Science,Cambridge, Maryland. 492 pp.
