# Supplementary material for: Seagrass contribution to blue carbon in a shallow karstic coastal area of the Gulf of Mexico
Source: PeerJ. 2021 Sep 10;9:e12109. doi: 10.7717/peerj.12109 (PMC8436957; doi:10.7717/peerj.12109)
Supplement: Supplemental Information 2 — Summary of seagrass community and structure measured in LPBR at different classes, showing mean ± SD, median, minimum and maximum values.Statistical data test (ANDEVA and Kruskall-wallis test). black letters represent statistical differences. [file peerj-09-12109-s002.docx]

Tabla 2.- Structural characteristics of seagrass meadows in by type of meadow within the reserve, the mean ± SD, median and minimum and maximum are shown.

| **Species** | **Meadow** | **n** | **ha** | **Total**  **Biomass**  **g Dw m^-2^** | **#shoots m^-2^** | **Coverage (%)** | **Macroalgae**  **Cover (%)** | **LAI** |
| --- | --- | --- | --- | --- | --- | --- | --- | --- |
| ***T. testudinum*** | **MxMa** | 36 | 51,884 |  |  |  |  |  |
| Mean± SE |  |  |  | 629±532 | 400±293 | 57±28 | 19±11 | 5±4 |
| Median |  |  |  | 469 | 294 | 50 | 17 | 2.6 |
| Min-Max |  |  |  | 47-1982 | 10-1235 | 15-100 | 5-40 | 0.1-14.4 |
| ***S. filiforme*** |  |  |  |  |  |  |  |  |
| Mean± SE |  |  |  | 367±253 | 732±513 | 46±23 |  |  |
| Median |  |  |  | 305 | 588 | 50 |  |  |
| Min-Max |  |  |  | 21-1010 | 144-2000 | 10-90 |  |  |
| ***H. wrightii*** |  |  |  |  |  |  |  |  |
| Mean± SE |  |  |  | 290±393 | 646±249 | 33±36 |  |  |
| Median |  |  |  | 123 | 646 | 15 |  |  |
| Min-Max |  |  |  | 35-988 | 470-823 | 10-75 |  |  |
| ***T. testudinum*** | **MxSf** | 14 | 44,492 |  |  |  |  |  |
| Mean± SE |  |  |  | 117±81 | 133±102 | 39±22 | 16±9 | 1.4±0.9 |
| Median |  |  |  | 81 | 103 | 37 | 12 | 1.2 |
| Min-Max |  |  |  | 14-248 | 47-382 | 15-100 | 10-30 | 0.24-3.14 |
| ***S. filiforme*** |  |  |  |  |  |  |  |  |
| Mean± SE |  |  |  | 120±86 | 364±370 | 28±13 |  |  |
| Median |  |  |  | 80 | 294 | 25 |  |  |
| Min-Max |  |  |  | 14-249 | 117-1000 | 15-60 |  |  |
| ***H. wrightii*** |  |  |  |  |  |  |  |  |
| Mean± SE |  |  |  | 271±254 | 823±513 | 26±15 |  |  |
| Median |  |  |  | 176 | 734 | 25 |  |  |
| Min-Max |  |  |  | 43-669 | 224-1596 | 10-50 |  |  |
| ***T. testudinum*** | **TtMa** | 22 | 30,189 |  |  |  |  |  |
| Mean± SE |  |  |  | 859±175 | 388±186 | 63±32 | 28±18 | 5±4 |
| Median |  |  |  | 859 | 344 | 70 | 20 | 2.66 |
| Min-Max |  |  |  | 735-982 | 112-794 | 10-100 | 10-55 | 0.6-15.7 |
| ***T. testudinum*** | **SfTt** | 14 | 23,048 |  |  |  |  |  |
| Mean± SE |  |  |  | 393±178 | 233±133 | 46±25 | 20±14 | 2.7±3 |
| Median |  |  |  | 332 | 240 | 35 | 20 | 1.9 |
| Min-Max |  |  |  | 195-717 | 25-470 | 15-100 | 10-30 | 0.2-10.9 |
| ***S. filiforme*** |  |  |  |  |  |  |  |  |
| Mean± SE |  |  |  | 266±289 | 531±446 | 56±33 |  |  |
| Median |  |  |  | 145 | 382 | 60 |  |  |
| Min-Max |  |  |  | 68-929 | 65-1470 | 10-100 |  |  |
| ***H. wrightii n=1*** |  |  |  |  |  |  |  |  |
| Mean± SE |  |  |  | 91 | 300 | 10 |  |  |
| Median |  |  |  |  |  |  |  |  |
| Min-Max |  |  |  |  |  |  |  |  |
